# Alopecia revealing a gastric trichobezoar in a child

**DOI:** 10.11604/pamj.2025.51.80.48705

**Published:** 2025-07-25

**Authors:** Leila Debono, Nour Mekaoui

**Affiliations:** 1 Pediatric Medical Emergency, Children's Hospital of Rabat, Rabat, Morocco,; 2 Faculty of Medicine and Pharmacy of Rabat, Mohamed V University in Rabat, Rabat, Morocco,; 3 Laboratory of Biostatistics, Clinical Research and Epidemiology, Faculty of Medicine and Pharmacy, Mohamed V University in Rabat, Rabat, Morocco

**Keywords:** Alopecia, trichobezoar, child

## Image in medicine

A 10-year-old girl, with no particular medical history, consulted the pediatric emergency room for alopecia and pallor observed by the mother for several months. The admission examination found a pale patient with mild alopecia over the frontal scalp but more pronounced at the eyebrows. The abdominal examination revealed a small, painless, and mobile epigastric mass, with no hepatomegaly or splenomegaly. The lymph node areas were free. Height and weight growth were normal according to age. The emergency abdominal ultrasound revealed that the mass corresponded to unidentified gastric contents, requiring additional computed tomography (CT) scanning. On the abdominal CT scan, the appearance was in favor of an endoluminal gastric mass, suggesting a trichobezoar. The history taken again in the young girl revealed trichophagia, of which the mother was unaware. The patient underwent surgical treatment after a transfusion due to the discovery of anemia, with good progress. The child was referred for psychiatric consultation for follow-up on her trichophagia.

**Figure 1:**
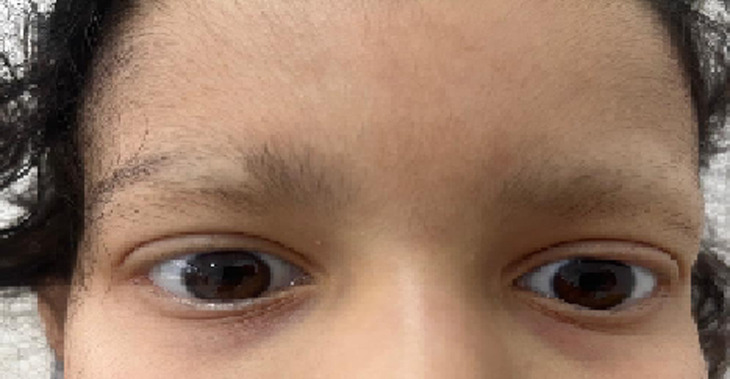
eyebrow alopecia that revealed a trichobezoar

